# Lateral single incision laparoscopic totally extraperitoneal hernioplasty (L-SILTEP) after laparoscopic radical prostatectomy: A rare case report with literature review

**DOI:** 10.1097/MD.0000000000034543

**Published:** 2023-08-11

**Authors:** Zhuoyin Wang, Qilei Li, Jiansong Tang, Farong Zhu, Yong Chen, Sen Lin, Yizhong Zhang

**Affiliations:** a Ningbo Beilun Third People’s Hospital, Ningbo, China; b The First Affiliated Hospital of Ningbo University, Ningbo, China.

**Keywords:** inguinal hernia, laparoscopic radical prostatectomy, L-SILTEP, SIL-TEP, Tep

## Abstract

Single-incision laparoscopic totally extraperitoneal hernioplasty is a commonly used surgical procedure for the treatment of inguinal hernia. However, it is difficult to use traditional single incision laparoscopic totally extraperitoneal hernioplasty to treat inguinal hernia after laparoscopic radical prostatectomy. We successfully and smoothly cured a patient with left inguinal hernia after laparoscopic radical prostatectomy using lateral single incision laparoscopic totally extraperitoneal hernioplasty.

**Case presentation:**

We report the case of a 70-year-old man who underwent laparoscopic radical prostatectomy 2 years earlier and had an evanescent mass in the left inguinal region for 1 month.

**Diagnosis:**

On the basis of preoperative abdominal computed tomography and intraoperative findings, the patient was diagnosed with a left indirect inguinal hernia, and post-laparoscopic radical prostatectomy.

**Interventions:**

The patient underwent lateral single incision laparoscopic totally extraperitoneal hernioplasty.

**Outcomes:**

The patient recovered well after the operation, and there were no postoperative complications or recurrence of inguinal hernia 3 months after the operation.

**Conclusion:**

For patients who have undergone laparoscopic radical prostatectomy, lateral single-incision laparoscopic totally extraperitoneal hernioplastycan be performed.

## 1. Introduction

An inguinal hernia occurs when tissues, such as part of the intestine, protrude through a weak spot in the abdominal wall near the groin area.^[1]^ The resulting bulge can be painful, particularly when 1 coughs, bends over, or lifts a heavy object. The risk factors for inguinal hernia include family history, previous contralateral hernia, male sex, age, abnormal collagen metabolism, prostatectomy, and low body mass index.^[2–4]^ It can be repaired surgically, either open or minimally invasive. Single incision laparoscopic totally extraperitoneal hernioplasty (SIL-TEP) is a single-incision laparoscopic totally extraperitoneal hernioplasty technique for repairing inguinal hernias using a single port. SIL-TEP has the advantages of less postoperative pain, faster recovery, and better cosmetic results than conventional laparoscopic or open surgery.^[5,6]^ However, it also has some limitations such as a narrow operative field, limited instrument maneuverability, and high technical difficulty.^[7,8]^ For patients with inguinal hernia after laparoscopic radical prostatectomy, it is difficult to perform SIL-TEP because the preperitoneal space in the traditional approach is the original surgical scar area, with serious adhesions and unclear boundaries. Lateral single incision laparoscopic totally extraperitoneal hernioplasty (L-SILTEP) is a modified technique that uses a lateral incision, instead of a central incision, to access the preperitoneal space. This allows for more space and better visualization of the anatomy, and reduces the risk of injury to the bladder or vessels. L-SILTEP has been reported to be feasible and safe for patients with recurrent or bilateral inguinal hernias,^[9]^ there are no reports on its application in patients after laparoscopic radical prostatectomy. This case report presents a surgical approach to lateral single-incision laparoscopic totally extraperitoneal hernioplasty after laparoscopic radical prostatectomy.

## 2. Case presentation

**Figure 1.**
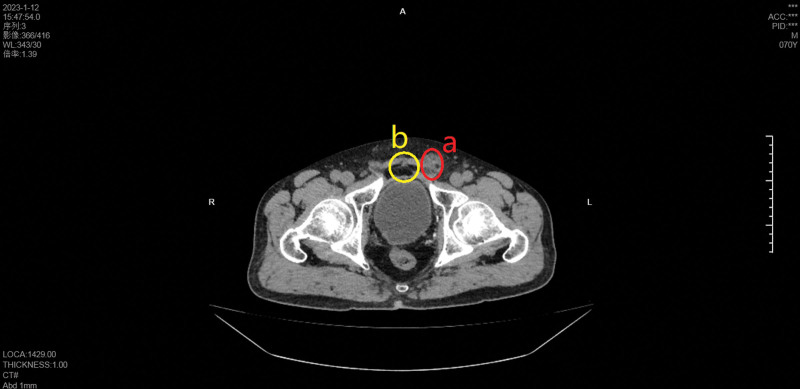
Computed tomography (CT). (A) The left inguinal hernia. (B) The scar of laparoscopic radical prostatectomy.

**Figure 2.**
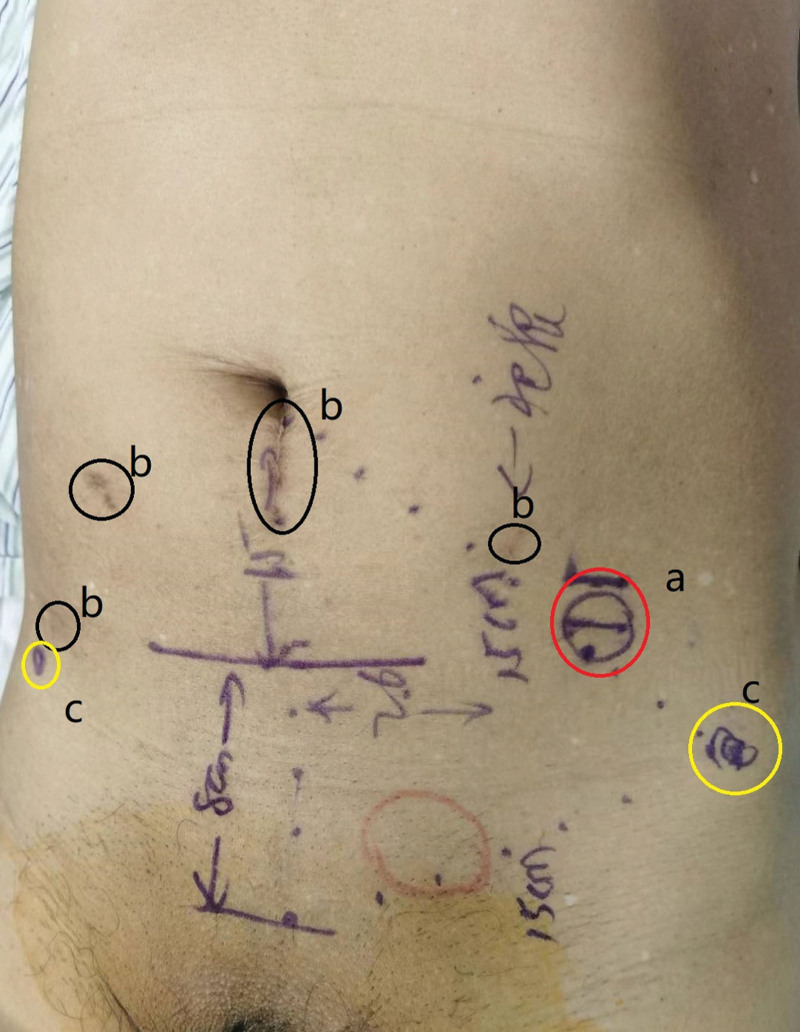
Surgical design. (A) Surgical incision. (B) The scar of laparoscopic radical prostatectomy. (C) Anterior superior iliac ridge.

The patient was a 70-year-old elderly male who underwent “laparoscopic radical prostatectomy + pelvic lymph node dissection” for “prostate cancer” 2 years ago for other reasons. Postoperative recovery was acceptable. However, he presented with an evanescent mass in the left inguinal region without an obvious cause 1 month prior, occasionally with pain, and with no exhaust or defecation disorder. The patient did not undergo regular treatment during this period. Physical examination: a mass measuring approximately 3.0 × 3.0 cm was palpable in the left inguinal region, which did not descend into the scrotum and disappeared when lying down. The mass was soft, without tenderness, and smooth on the surface. After reduction, the patient was instructed to cough, and a finger could feel an impulse. The finger pressed the deep ring and instructed the patient to stand up and cough; the mass did not protrude. The finger was removed and the mass protruded. Laboratory test results were normal. Abdominal computed tomography revealed a left inguinal hernia and postoperative prostate changes. We comprehensively evaluated the patient inguinal hernia and laparoscopic postoperative abdominal adhesions of prostate cancer before surgery (Fig. 1). The lower part of the patient umbilicus was the incision of laparoscopic radical prostatectomy, approximately 5 cm long, which was postoperative scar tissue that interfered with the traditional SIL-TEP surgery through the lower umbilical margin incision, affecting the separation of tissues and the placement of patches during surgery. Therefore, after careful discussion and excellent design, we designed a lateral-approach SILTEP surgery (L-SILTEP) for this patient (Fig. 2).

The following are the specific surgical procedures.

**Figure 3.**
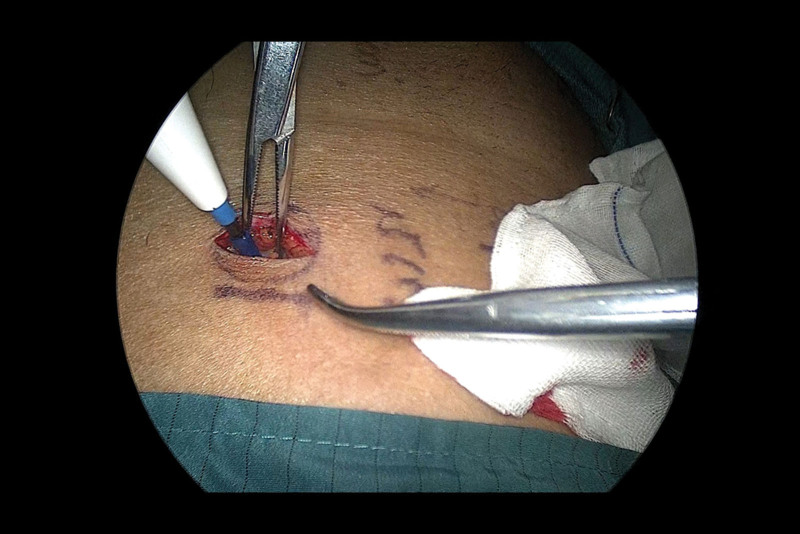
Surgical incision.

**Figure 4.**
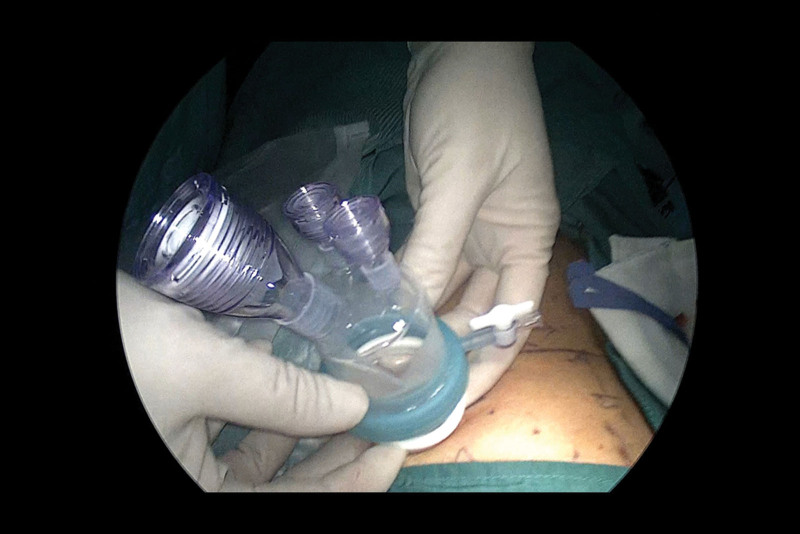
The single-port incision device.

First, the incision was selected and the instrument was placed. A 2-cm incision was made along the skin crease approximately 1 cm above the McBurney point on the left side (Fig. 3), the skin and subcutaneous tissue were incised, and an oblique aponeurotic incision was made in the direction of the aponeurosis of the external oblique muscle. The internal oblique and transverse abdominal muscles were bluntly separated to expose the peritoneal fat, and the preperitoneal space was dilated using the index finger. We then inserted a single-port incision device, established pneumoperitoneum, and set the pneumoperitoneum pressure to 12 mm Hg (Fig. 4).

**Figure 5.**
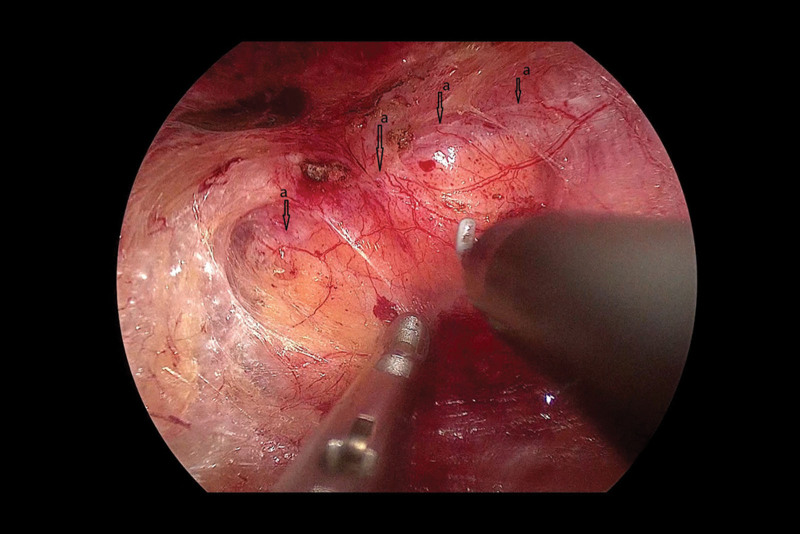
At the beginning of the operation, we found a dense adhesion of the peritoneum to the posterior sheath of the rectus abdominis muscle. (A) The peritoneum densely adhered to the posterior sheath of the rectus abdominis.

**Figure 6.**
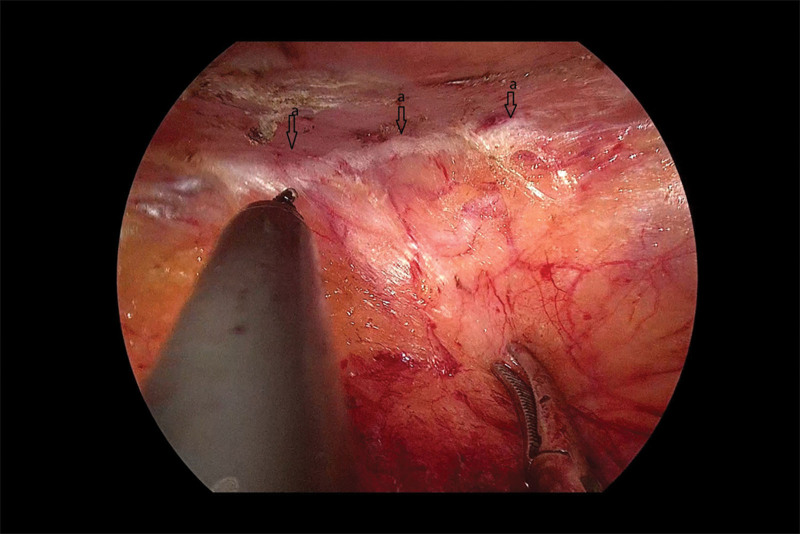
(A) The densely adhered Retzius space.

Next, we established a tract and separated the space. We cut through the extraperitoneal fat layer along the upper edge of the internal ring to enter the Bogros space, and observed that the Bogros space area was naturally loose. We continued to use the electrocoagulation hook to separate along the direction of the internal ring mouth, with the peritoneum as a guide, and reached the lower abdominal vessels. We observed that the peritoneum and the posterior sheath of the rectus abdominis were tightly adhered (Fig. 5) and carefully separated the adherent peritoneum from the front of the Retzius space using a sharp-blunt alternating electrocoagulation hook. We saw that the Retzius space was densely adhered (Fig. 6), and initially expanded the Retzius space, and then separated the surrounding space of the hernia sac in an outward and downward direction. We continued to separate until we initially established sufficient space to deal with the hernial sac.

**Figure 7.**
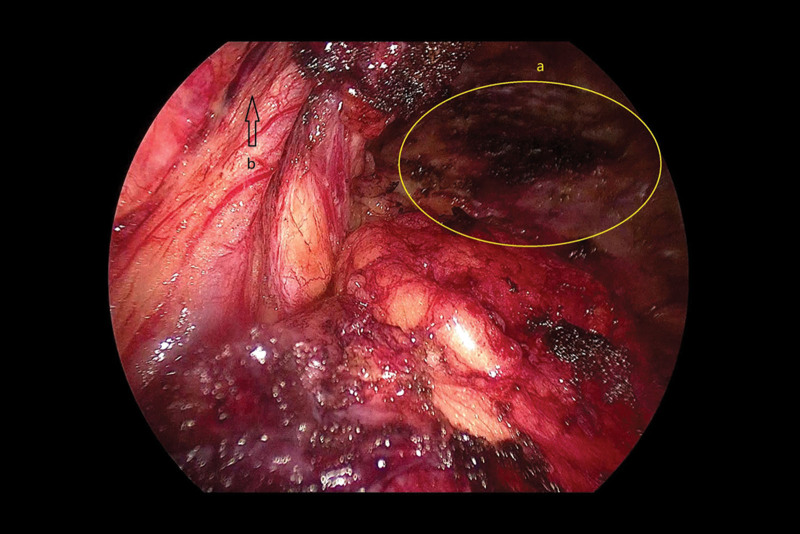
(A) The separation of the completed Retzius space. (B) Spermatic cord vessels.

**Figure 8.**
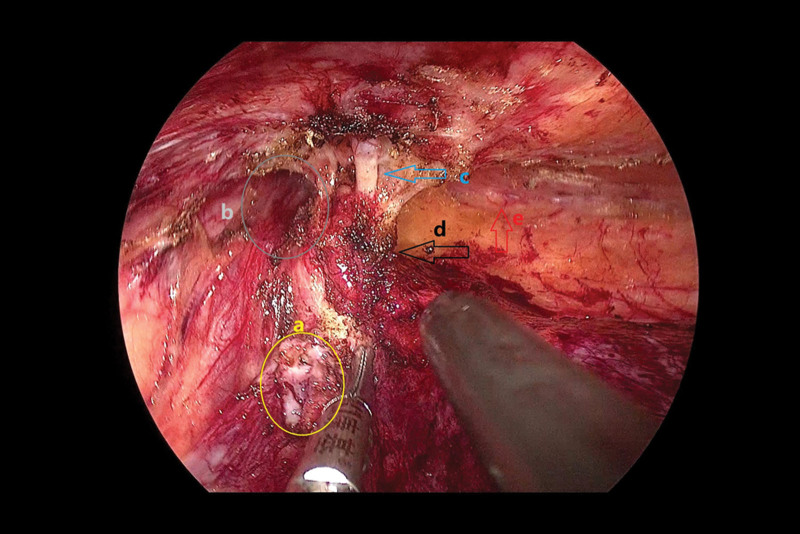
(A) Severed hernia sac. (B) The inguinal canal. (C) Inferior epigastric artery. (D) The hernia sac around the surrounding dense adhesions. (E) Pectineal ligament.

Next, we examined the hernial sac. We found dense adhesions around the hernia sac and first separated the hernia sac from the lateral posterior side along the lateral peritoneum, freeing up the spermatic cord vessels. Then, we turned to the inner side of the internal ring to extend the space and separated it from the anterior inner side. After carefully identifying the relationship between the hernial sac and abdominal wall vessels, we found that the hernial sac was located on the lateral side of the abdominal wall vessels. Therefore, the intraoperative diagnoses were as follows: Left indirect inguinal hernia; Post-laparoscopic radical prostatectomy. The hernial sac was transected and ligated along the distal direction of the internal ring. The spermatic cord structure was de-peritonealized at a position >6 cm above the internal ring and flattened the peritoneal fold line. We further expanded the Bogros space along the inner side of the iliopubic tract until it reached the anterior superior iliac spine. We continued to deal with the Retzius space, and by careful traction and electrocoagulation, we separated it to a position 2 cm past the pubic symphysis distally, then enlarged the space from bottom to top, reaching 8 cm above the pubic symphysis and 2 cm below the pubic comb ligament (Figs. 7 and 8).

**Figure 9.**
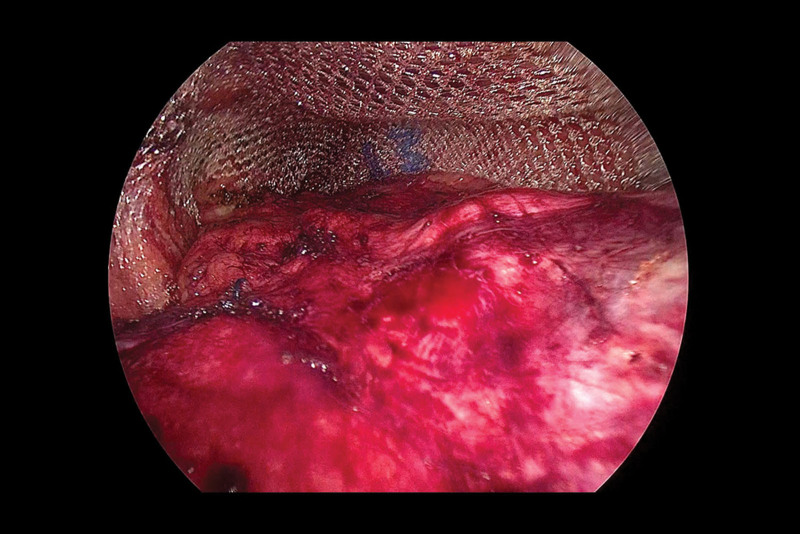
Mesh placement was completed.

Then we placed a patch. We used a large mesh lightweight 3DMax patch (large size), rolled up the patch internally, and placed it through the middle observation hole of the single-port device, centered on the hernia ring and unfolded patch, completely covering the myopectineal orifice. Patch laying adopted the method of first inner lower than outer upper layer. We then used separation forceps to press down the lower edge of the patch under direct vision exhaust while observing the peritoneum lifting the pressing patch (Fig. 9). The port was removed and the incision was checked for bleeding. The internal oblique muscle fascia was sutured and the cut external oblique muscle fascia was continuously sutured. The skin was then sutured to close the incision.

**Figure 10.**
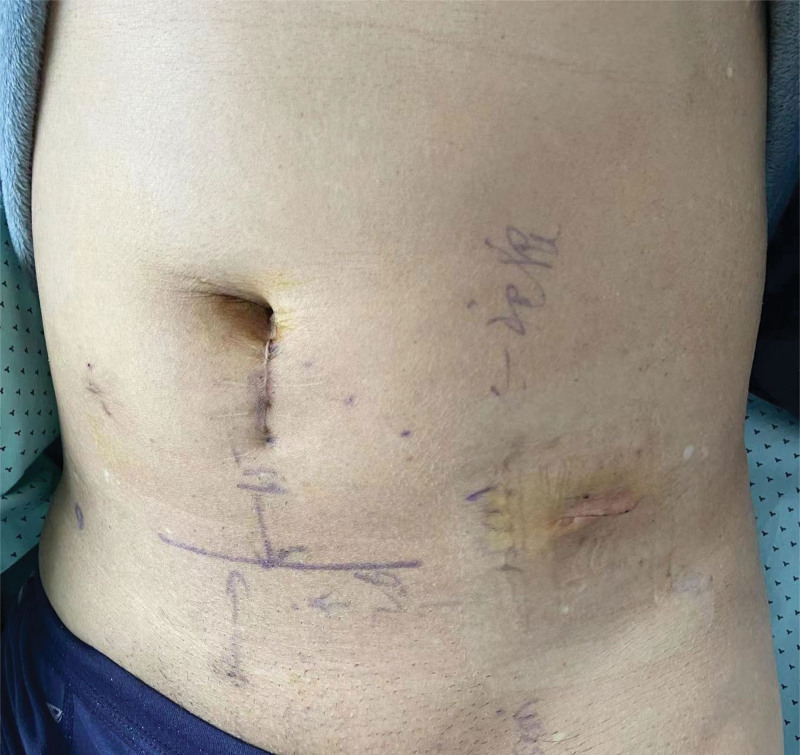
First postoperative day.

The patient recovered smoothly after the surgery. The patient started walking on the ground 6 hours after surgery (Fig. 10). The patient was discharged 2 days after surgery. At the time of writing this article (three months after surgery), there was no recurrence, the wound healed well, and there were no postoperative complications. The patient expressed great satisfaction with the results of the operation.

## 3. Discussion

An inguinal hernia after laparoscopic radical prostatectomy is a rare but challenging condition. Studies have shown that inguinal hernia is a common late complication of radical prostatectomy, with an incidence of 12% to 25%.^[10,11]^ This may be due to the intraoperative disruption of the anatomy supporting the inguinal region or changes in intra-abdominal pressure after prostatectomy. The risk of inguinal hernia repair after radical prostatectomy varies according to the type of prostatectomy. Some studies have suggested that the risk of inguinal hernia formation and repair by minimally invasive techniques such as laparoscopic or robot-assisted surgery may be lower than that of open surgery, while others have found no significant difference.^[11–13]^ It is difficult to repair inguinal hernia after laparoscopic radical prostatectomy, either by open repair or laparoscopic repair, because of the severe adhesion of the preperitoneal space and unclear anatomy. In addition, the traditional laparoscopic technique has been abandoned by most surgeons because of the possibility of manipulation.

L-SILTEP is a novel technique that uses a lateral rather than a central incision to access the preperitoneal space. On the one hand, surgical scar areas around or below the umbilical cord can be completely avoided. In contrast, the external oblique, internal oblique, and transversus abdominis muscles of the external abdominal wall were not cut, and different directions of the 3 layers of flat muscles were used to reduce the incidence of incisional hernia after surgery. During the operation, the extraperitoneal fat can be cut directly into the Bogros space, and the operation can be performed along the pre-peritoneum under the guidance of the peritoneum, avoiding the complex structure of the transversalis fascia in the lower abdomen. Dissection was performed within or between the multilayer preperitoneal fascia, and the anatomy was relatively simple. For indirect hernia, especially scrotal hernia, the approach from Bogros’ space to the peritoneum straight into the inner ring makes it easier to expose and separate the hernia sac, simplifying the surgical procedure and reducing the bleeding at the surgical level. It has been reported that L-SILTEP is safe and feasible in patients with recurrent or bilateral inguinal hernia; however, its application in patients after laparoscopic radical prostatectomy has not been reported.

In summary, we reported a case of L-SILTEP for inguinal hernia after laparoscopic radical prostatectomy. L-SILTEP is a feasible option for the treatment of such patients, especially those with a history of lower umbilical incisions. However, more studies are needed to compare the efficacy and safety of L-SILTEP with other techniques for the treatment of inguinal hernia after laparoscopic radical prostatectomy.

## Author contributions

**Conceptualization:** Zhuoyin Wang, Qilei Li, Yong Chen, Yizhong Zhang.

**Investigation:** Zhuoyin Wang, Qilei Li, Farong Zhu, Yong Chen, Sen Lin.

**Methodology:** Zhuoyin Wang, Jiangsong Tang.

**Supervision:** Zhuoyin Wang, Farong Zhu, Sen Lin, Yizhong Zhang.

**Validation:** Zhuoyin Wang, Jiangsong Tang, Yizhong Zhang.

**Writing – original draft:** Zhuoyin Wang.

**Writing – review & editing:** Zhuoyin Wang, Yizhong Zhang.

## References

[1] Shakil A Aparicio K Barta E. Inguinal hernias: diagnosis and management. Am Fam Physician. 2020;102:487–92. PMID: 33064426. 33064426

[2] LeBlanc KE LeBlanc LL LeBlanc KA. Inguinal hernias: diagnosis and management. Am Fam Physician. 2013;87:844–8. PMID: 23939566. 23939566

[3] Öberg S Andresen K Rosenberg J. Etiology of inguinal hernias: a comprehensive review. Front Surg. 2017;4:52. PMID: 29018803; PMCID: PMC5614933. 29018803 10.3389/fsurg.2017.00052 PMC5614933

[4] HerniaSurge Group. International guidelines for groin hernia management. Hernia. 2018;22:1–165. Epub 2018 Jan 12. PMID: 29330835; PMCID: PMC5809582. 10.1007/s10029-017-1668-x PMC5809582 29330835

[5] Park YY Lee K Oh ST. Learning curve of single-incision laparoscopic totally extraperitoneal repair (SILTEP) for inguinal hernia. Hernia. 2022;26:959–66. Epub 2021 Jun 7. PMID: 34097186. 34097186 10.1007/s10029-021-02431-7

[6] Cardinali L Mazzetti CH Cadenas Febres A. Prospective randomized study comparing single-incision laparoscopic versus multi-trocar laparoscopic totally extraperitoneal (TEP) inguinal hernia repair at 2 years. Surg Endosc. 2018;32:3262–72. Epub 2018 Jan 23. PMID: 29362907. 29362907 10.1007/s00464-018-6045-z

[7] Kim JH An CH Lee YS. Single incision laparoscopic totally extraperitoneal hernioplasty (SIL-TEP): experience of 512 procedures. Hernia. 2015;19:417–22. Epub 2014 Dec 24. PMID: 25537571. 25537571 10.1007/s10029-014-1337-2

[8] Zhou E Qi C Wang X. Single incision laparoscopic totally preperitoneal hernioplasty (SIL-TPP): lessons learned from 102 procedures and initial experience. Medicine (Baltim). 2022;101:e30882. PMID: 36181025; PMCID: PMC9524943. 10.1097/MD.0000000000030882 PMC9524943 36181025

[9] Lee JH Kim YJ Kim WJ. Single-incision laparoscopic totally extraperitoneal inguinal hernia repair using an innovative transabdominal preperitoneal approach: initial experience and early outcomes. Surg Endosc. 2012;26:3405–10.

[10] Miyajima A. Inseparable interaction of the prostate and inguinal hernia. Int J Urol. 2018;25:644–8. Epub 2018 Jun 19. PMID: 29923274. 29923274 10.1111/iju.13717

[11] Ahtinen M Vironen J Murtola TJ. The risk of inguinal hernia repair after radical prostatectomy - a population-based cohort study. Scand J Urol. 2022;56:191–6. Epub 2022 Apr 22. PMID: 35451920. 35451920 10.1080/21681805.2022.2065357

[12] Liu L Xu H Qi F. Incidence and risk factors of inguinal hernia occurred after radical prostatectomy-comparisons of different approaches. BMC Surg. 2020;20. 10.1186/s12893-020-00883-9 PMC7532612 33008371

[13] Nilsson H Stranne J Hugosson J. Risk of hernia formation after radical prostatectomy: a comparison between open and robot-assisted laparoscopic radical prostatectomy within the prospectively controlled LAPPRO trial. Hernia. 2022;26:157–64. Epub 2020 Apr 11. Erratum in: Hernia. 2022 Apr;26(2):683. PMID: 32279170; PMCID: PMC8881255. 32279170 10.1007/s10029-020-02178-7 PMC8881255

